# Minimally required essential bio markers for lymphoma

**DOI:** 10.6026/973206300220333

**Published:** 2026-01-31

**Authors:** Shikha Ghanghoria, Arvind Ghanghoria, Gulladurthi Prudhvi Sai Chownika, Aashita Thakur, Tarun Prakash Verma, Sachin Parmar

**Affiliations:** 1 Department of Pathology MGMMC MYH, Indore, Madhya Pradesh, India; 2 Department of General Surgery MGMMC MYH, Indore, Madhya Pradesh, India; 3 Multidisciplinary Research Unit, MGM Medical College Indore, Madhya Pradesh, India; 4 Department of Community Medicine, V.K.S. Government Medical College, Neemuch, Madhya Pradesh, India

**Keywords:** Lymphoma, immunohistochemistry, Ki-67, B-cell markers, t-cell markers

## Abstract

Lymphoma is a diverse group of blood cancers arising from lymphoid tissue, characterized by distinct morphological, immunophenotypic, genetic and clinical variations. Therefore, it is of interest to identify which immunohistochemical (IHC) markers were minimum essentials markers to enable classification and prognostication in lymphoid malignancies. The retrospective study used a primary panel of monoclonal antibodies consisting of CD3 and CD5, CD20, CD10, CD45, PAX-5 and Ki-67 on 88 lymphoma cases. It was found that B-cell lymphomas were highly labeled with CD20, CD45, PAX-5, whereas T-cell lymphomas were heavily labeled with CD3, CD5. There were high levels of Ki-67 proliferation indices in the aggressive lymphoma sub-types e.g. Diffuse Large B-Cell Lymphoma and T-cell lymphomas. The study helps to recommend a restricted yet cost-effective panel of IHC markers to attain reliable diagnosis and prognosis of lymphoma particularly in lower resource settings.

## Background:

Lymphoma is a heterogeneous group of malignant neoplasms of hematopoietic tissue originating in lymphoid tissue and with interspecific morphological, immunophenotypic, genetic and clinical features. As we made progress in our knowledge of the biology of lymphoma and developed more advanced diagnostic techniques, it has become ever more central to determine precisely the subtype of lymphoma and to do that as soon as possible in order to institute therapy and prognosis. However, one of the most important diagnostic modalities is immunohistochemistry (IHC) because of its capacity to identify the cellular lineage, state of maturity and even genetic aberration with the help of antibodies to stain cell lineage and disease-associated markers [1, 2]. Initial assessment of a suspected lymphoma is a conglomeration of clinical history, radiological examination, morphological and immunofenotyping. Morphological features in isolation, however, are frequently not adequate to distinguish between different subtypes of lymphoma as there may be overlap of the characteristics with reactive or other neoplastic processes [1, 3]. Thus, a lean but efficient IHC marker panel should always be employed in order to achieve a definitive diagnosis especially in resource-limited or limited access to large marker panel [3, 4 and 5]. In case of B-cell lymphomas, CD20, a pan B-cell marker, in most B-cell neoplasms, is usually included along with CD3, a pan T-cell marker, as a negative control of B-cell lineage [5, 6]. In order to identify clonality, kappa and lambda light chains may be seen to be essential as well, particularly in patients that exhibit similarities to chronic lymphocytic leukemia/ small lymphocytic lymphoma (CLL/SLL). Other markers such as CD5 and CD23 assist in the distinction between CLL/SLL, mantle cell lymphoma and follicular lymphoma and cycling D1 is paramount in the diagnosis of mantle cell lymphoma [6, 7]. Follicular lymphoma is characterized by expression of Bcl-2 and CD10 and aggressiveness can be graded on the proliferating area [1, 5]. The T-cell and NK-cell lymphomas are attended by the utilization of universal T-cell antigens like CD3 and CD2 and also CD5, CD7, CD4 and CD8 to further characterize the lineage subtypes [6, 7]. Relevant cases will be added with markers CD56 and cytotoxic markers (granzyme B, TIA-1) with NK-cells. In classical Hodgkin lymphoma, the most typical targets of Reed-Sternberg cells are CD30 and CD15 and they are frequently accompanied by CD20, CD3 and PAX-5 to confirm the lineage [2, 7]. Furthermore, the ambiguous cases can require additional supporting studies (like in situ hybridization of EBV-encoded RNA, molecular, or cytogenetic procedures) which are not generally available [2, 3 and 8]. However, the minimal panel which was mentioned above is the key to a practical, cost-effective and reliable strategy to most lymphoma diagnoses. The sensible choice and interpretation of such markers incorporated in clinical and morphologic backdrop steers clinicians and pathologists to the right classification and best treatment of lymphoma patients [1, 3 and 5]. Our study demonstrates that a limited, resource- feasible IHC panel (CD3, CD20, Ki 67 with targeted add-on markers such as PAX5/CD79a when needed) can reliably establish lineage and support WHO-aligned subtyping in most lymphoma cases, aligning with recommended diagnostic panel approaches. Therefore, it is of interest to document the Minimally Required Essential Bio Markers for Lymphoma.

## Methods and Materials:

The Minimal Essential Markers to Reach Lymphoma is a study done in Multidisciplinary research unit department of health research government of India at Maharaja Yashwantrao Hospital and Mahatma Gandhi Government Medical College, a Tertiary Care Hospital in Madhya Pradesh, India. The study was meant to determine minimal essential markers in the classification and prognostication of lymphoid malignancies. Eighty-eight lymphoid malignancies were diagnosed during the two years and morphological examination was initially used to make the diagnosis.

## Study design:

It is an observational, retrospective study that will involve cases diagnosed with lymphoid malignancies in the medical facilities listed above during the period between January 2021 and December 2022. Each case was accessed and categorized using the set histopathological and immunohistochemical standards.

## Patient selection:

Of the patients who participated in the study, 88 were diagnosed with a wide range of lymphoid malignancies over the study period. The inclusion criteria included all age and gender patients of the patients diagnosed with lymphoid malignancies according to the primary morphological assessments. Inadequate sample tissue for immunohistochemical analysis was used as an exclusion criterion.

## Immunohistochemical analysis:

The lymphoid malignancies were further classified using a primary panel of monoclonal antibodies applied to the tissue samples. The following markers were used in this study:

CD3, CD5: T-cell markers used in the identification of T-lymphocytes.

CD10: An antigen employed to determine the presence of follicular center B-cells.

CD15, CD20: Bells markers indicating the presence of mature B-cells.

CD23: An identifier of follicular lymphoma.

CD30: A marker that identifies Hodgkin lymphoma and anaplastic large cell lymphoma.

PAX-5: A B-cell marker that is used to identify B-cell lineage.

Tote (Terminal deoxynucleotidyl transferals): A precursor marker of early T-cell and B-cell.

CD45: The standard leukocyte antigen to verify that the cells were lymphoid.

CyclinD1: A marker to detect mantle cell lymphoma.

Bcl-2: A probe to identifying anti-apoptotic proteins linked with different subtypes of lymphoma.

The immunohistochemical stainingwas carried out with the usual methods on formalin-fixed and paraffin-embedded histology sections. The antibodies were used as per the recommendation of the manufacturer and the relevant positive and negative controls were also included to make sure that the results would be valid.

## Ki-67 proliferative index:

The Ki-67 marker was applied to evaluate the proliferative activity status of the lymphoma cells. Ki-67 positive cells were quantified as percentage in each case and the proliferative index was computed. It was tested, as a prognostic marker, whereby elevated Ki-67 indicated an increased aggressive form of lymphoma.

## Final classification:

The cases were all categorized with the world health organization (WHO) classification of lymphoid neoplasms. This system of classification combines morphological andimmuno phenotypicinformation to identify the type of lymphoma.

## Statistical analysis:

The free online available statistical calculator was used to analyze data. Frequencies and percentages were calculated as descriptive statistics to provide a description of the distribution of cases by lymphoma subtype. Pearson correlation coefficient was used to study the relationship between Ki-67 proliferation index and prognosis. Statistically significant was a p-value of less than 0.05.

## Ethical considerations:

The institutional ethics committee of Maharaja Yashwantrao Hospital and Mahatma Gandhi Government Medical College was granted permission to conduct the research. Data were anonymized and informed consent was sought when necessary to ensure patient confidentiality.

## Results:

Among 88 lymphoma cases over 2 years, Non Hodgkin lymphoma (NHL) constituted 84.1% and Hodgkin lymphoma (HL) 15.9%, with B cell lymphomas more frequent than T cell lymphomas (75.6% versus 24.4%); this lineage allocation was supported on immunophenotyping using a core panel (CD3, CD5, CD20, CD45, PAX 5) showing predominant CD20/CD45/PAX 5 positivity in B cell neoplasms and CD3/CD5 positivity in T cell neoplasms (Table 1-Table 2). Within NHL, the major B cell subtypes included DLBCL (20.5%), follicular lymphoma (11.3%), MALT lymphoma (11.3%), SLL/CLL (6.8%), mantle cell lymphoma (4.5%), high grade B cell lymphoma (6.8%) and T cell/histiocyte rich large B cell lymphoma (2.3%), each demonstrating the expected subtype- specific marker patterns and Ki 67 score ranges (Table 1). T cell/NK-cell neoplasms were mainly adult T cell lymphoma (18.3%) with a smaller proportion of extranodal T cell lymphoma (2.3%), strongly expressing CD3, CD5 and CD45 with consistently high Ki 67 (score 3) (Table 2). Classical HL comprised mixed cellularity (11.3%), nodular sclerosis (2.3%) and lymphocyte-rich (2.3%) subtypes, all showing CD15 and CD30 positivity with low Ki 67 scores (0-1) (Table 3). Ki 67 expression varied significantly across entities (p = 0.0017), with universal positivity in DLBCL, high grade B cell lymphoma and T cell lymphomas, whereas indolent B cell lymphomas showed lower Ki 67 positivity; correspondingly, low Ki 67 scores clustered in indolent B cell lymphomas and all HL cases, while high scores were restricted to aggressive B cell and T cell lymphomas (Table 4- Table 5).

## Discussion:

The minimum requisite immunohistochemistry (IHC) markers required to diagnose lymphoma continues to be one of the topics of widespread discussion and acceptance in the literature of pathology. The present report confirms the importance of indicators CD20 and CD3 to determine the lineage and CD79a is a valid substitute to CD20 where the expression is changed as a result of treatment or differentiation of the tumor cells. This is quite similar to what Cho lifts where a staining of the CD3 (T-cell marker), CD20 (B-cell markers) and the other markers such as Ki-67, CD30, CD15 and also PAX5 are considered as basic especially in both Hodgkin lymphoma and non-Hodgkin lymphoma [9]. Remarkably, Isanti and colleagues considered more than 800 cases of possible lymphomas in order to determine the usefulness of minimally reduced IHC panels in B-cell lymphoma. They verified that even panels as small as three to five antibodies would enable proper diagnosis in around 70 percent of cases with the remaining requiring additional markers or molecular analysis. Their observations support the significance of a definite diagnostic algorithm, especially in environments with limited resources, which are similar to the key outcomes of the current findings [5]. Similarly, Nisha *et al.* propose a two-t tier strategy, suggesting a simple panel of CD5, CD23, CD79a and light chains (Kappa, lambda) as an initial primary screen, with additional panels to more delicately subtype B-cell non-Hodgkin lymphoma. Their method also effectively discriminates CLL/SLL, mantle cell lymphoma and follicular lymphoma, which is in agreement with the marker selection strategy presented in the current research with regard to markers [10]. In the case of mature B-cell lymphomas (including follicular lymphoma and Burkitt lymphoma), Huan-You *et al.* recommend minimal panel comprising BCL2, CD3, CD10 and CD20 (more recent updates on this staining-based gradation emphasize the usefulness of germinal center- related markers as additional subtyping and grading of the disease). They highlight the fact that Ki-67 is needed to assess proliferation, which is also done in various guidelines, as is evident in the study design too [11]. The study on concordance between IHC and molecular testing (MT) by Maneesh *et al.* involved a comparative assessment that resulted in high concordance rates in the classification of subtypes: e.g., IHC and MT classified DLBCL in 37.5 percent and 40 percent, respectively. But there were rare incidences in which absence was noted in unusual subtypes such as Burkitt lymphoma, with 2 to 7 percent demonstrating in discrepancies [12].

## Conclusion:

There is a persistent call amongst IHC panel preparation to champion a focused-lineage focused and proliferation focused panel that is functional as well as a powerful diagnostic correlate. Naturally, slight variations in panel composition may relate to case mix, resources available and changing disease taxonomy; however, the key markers provided by the international literature largely confirms the marker selection strategy of the present review.

## Figures and Tables

**Table 1:** Distribution of NHL subtypes and marker profiles

**Subtype**	**% of Total**	**Immunophenotypic Markers**	**Ki-67 Score**
Diffuse Large B-Cell Lymphoma (DLBCL)	20.50%	CD45+, CD20+, CD10+, CD5 (variable), PAX-5+	Score 2-3
Follicular Lymphoma	11.30%	CD20+, CD10+, CD45+, PAX-5+, Bcl-2+	Score 0-2
Extranodal Marginal Zone Lymphoma (MALT)	11.30%	CD20+, PAX-5+, CD45+	Score 0-2
Small Lymphocytic Lymphoma (SLL/CLL)	6.80%	CD5+, CD20+, CD23+, CD45+, PAX-5+	Score 0-2
Mantle Cell Lymphoma (MCL)	4.50%	CD5+, CD20+, CD45+, PAX-5+, Cyclin D+	Score 0-2
High-Grade B-Cell Lymphoma (Extranodal)	6.80%	CD10+, CD20+, CD45+	Score 3
T-cell/Histiocyte-Rich Large B-Cell Lymphoma (TCRLBCL)	2.30%	CD3+, CD20+	Score 2

**Table 2:** T-cell and NK-cell neoplasms marker profile

**Subtype**	**% of Total**	**Immunophenotypic Markers**	**Ki-67 Score**
Adult T-cell Lymphoma	18.30%	CD3+, CD5+, CD45+	Score 3
Extranodal T-cell Lymphoma	2.30%	CD3+, CD5+, CD45+	Score 3

**Table 3:** Classical Hodgkin lymphoma subtypes and marker expression

**Subtype**	**% of Total**	**Immunophenotypic Markers**	**Ki-67 Score**
Mixed Cellularity	11.30%	CD15+, CD30+	Score 0-1
Nodular Sclerosis	2.30%	CD15+, CD30+	Score 0-1
Lymphocyte-Rich	2.30%	CD15+, CD30+	Score 0-1

**Table 4:** Ki-67 Positivity in NHL Subtypes

**Subtype**	**Ki-67 Positive**	**Ki-67 Score**
DLBCL	18/18	Score 2-3
SLL/CLL	6-Jun	Score 0-2
Mantle Cell Lymphoma	4-Feb	Score 0-2
Follicular Lymphoma	10-Jun	Score 0-2
TCRLBCL	2-Feb	Score 2
MALT Lymphoma	10-Oct	Score 0-2
High-Grade B-cell Lymphoma	6-Jun	Score 3
Adult T-cell Lymphoma	16/16	Score 3
Extranodal T-cell Lymphoma	2-Feb	Score 3

**Table 5:** Ki-67 score distribution by lymphoma category

**Score Category**	**B-cell Lymphomas**	**T-cell Lymphomas**	**Hodgkin Lymphoma**	**Others**
Low (0-15%)	15	-	14	-
Intermediate (16-30%)	27	-	-	-
High (>30%)	14	18	-	-

## References

[1] Swerdlow SH (2016). Blood..

[2] Rao SI (2010). Indian journal of medical and paediatric oncology..

[3] Coiffier B (2002). The New England journal of medicine..

[4] Falini B (1999). Blood..

[5] Ott G (2013). Blood..

[6] Disanto MG (2016). American journal of clinical pathology..

[7] Hans CP (2004). Blood..

[8] Adil SAK (2025). J Med Sci Res..

[9] Junhun C (2022). Blood research..

[10] Nisha M (2021). Blood research..

[11] Huan-You W, Zu Y (2017). Archives of pathology & laboratory medicine..

[12] Maneesh R (2024). Int J Med Public Health..

